# Handgrip strength asymmetry as a new biomarker for sarcopenia and individual sarcopenia signatures

**DOI:** 10.1007/s40520-023-02539-z

**Published:** 2023-09-02

**Authors:** Jedd Pratt, Ludmilla Pessanha, Marco Narici, Colin Boreham, Giuseppe De Vito

**Affiliations:** 1 Institute for Sport and Health, University College Dublin, Dublin, Ireland; 2 Department of Biomedical Sciences, Neuromuscular Physiology Laboratory, CIR-Myo Myology Centre, University of Padova, Padua, Italy; 3 Conway Institute of Biomolecular and Biomedical Research, University College Dublin, Dublin, Ireland

**Keywords:** Maximal strength, Strength asymmetry, Sarcopenia, Screening, Biomarkers

## Abstract

**Background:**

Although handgrip strength (HGS) asymmetry has clinical screening utility, its relevance to sarcopenia is unknown. This study examined the relationship between HGS asymmetry and sarcopenia signatures, and explored the relevance of circulating neural/neuromuscular markers.

**Methods:**

9403 individuals aged 18–92 years participated in this study. Maximal HGS and skeletal muscle index (SMI) were determined using hand dynamometry and DXA. Sarcopenia was diagnosed upon the presence of low HGS and low SMI, according to cohort-specific thresholds. Plasma biomarkers were measured by ELISA in a sub-group of 269 participants aged 50–83 years. Asymmetry was determined as the highest recorded HGS divided by the highest recorded HGS of the opposite hand. Individuals with a ratio > 1.10 were classified as having asymmetrical HGS.

**Results:**

Subjects with asymmetrical HGS had significantly lower SMI (7.67 kg/m^2^ vs 7.71 kg/m^2^, *p* = 0.004) and lower HGS (37.82 kg vs 38.91 kg, p < 0.001) than those with symmetrical HGS. In those aged ≥ 50 years asymmetrical HGS was associated with 2.67 higher odds for sarcopenia [95% confidence interval: (CI) = 1.557–4.561, p < 0.001], 1.83 higher odds for low HGS only (CI 1.427–2.342, p < 0.001), and 1.79 higher odds for low SMI only (CI 1.257–2.554, *p* = 0.001). HGS asymmetry demonstrated acceptable diagnostic accuracy for sarcopenia (AUC = 0.727, CI 0.658–0.796, p < 0.001). Plasma neural cell adhesion molecule concentrations were 19.6% higher in individuals with asymmetrical HGS (185.40 ng/mL vs 155.00 ng/mL, p < 0.001) than those with symmetrical HGS.

**Discussion:**

Our findings demonstrate the utility of HGS asymmetry as a screening tool that may complement existing strategies seeking to combat sarcopenia. Biomarker analyses suggest that heightened denervation may be an important aetiological factor underpinning HGS asymmetry.

**Supplementary Information:**

The online version contains supplementary material available at 10.1007/s40520-023-02539-z.

## Introduction

Handgrip strength (HGS) is a simple, well-established measure of muscle strength that robustly correlates with a myriad of important health variables [1–5]. Most striking perhaps, is the powerful predictive capacity of HGS for future disability [6], morbidity [7] and all-cause mortality [8]. The distinct clinical relevance coupled with ease of administration has led HGS assessment to be widely implemented as a screening tool in public health settings. Notably, HGS is one of the primary diagnostic criteria for sarcopenia, the age-related loss of muscle mass and function that affects over 40% of people aged ≥ 80 years [9, 10].

Presently, HGS determination protocols focus predominantly on the assessment of maximal HGS [11, 12]. Although maximal HGS has undoubted clinical relevance, and represents a suitable proxy for overall muscle strength, it does not provide context on another crucially important domain underpinning physical function, that of muscle strength symmetry [13, 14]. Importantly, evidence suggests that muscle strength asymmetry may signal deteriorating muscle health prior to a decline in maximal muscle strength [15]. In this regard, HGS asymmetry, commonly classified as a > 10% difference between hands [6, 15, 16], may represent a more sensitive and clinically relevant screening tool for muscle health than maximal HGS alone. Although recent data suggest that HGS asymmetry is an important predictor for falls and physical dysfunction [6, 17], the relationship between HGS asymmetry and clinical manifestations of sarcopenia has yet to be elucidated. In this regard, determining the relevance of HGS asymmetry to sarcopenia may lead to an enhancement of the prognostic value of HGS. Moreover, given that the assessment of left and right HGS is already included in current HGS protocols [11, 12], HGS asymmetry determination could be conveniently implemented in routine public health assessments. As such, broadening current HGS assessment protocols to include an interpretation of asymmetry may represent an efficient means of gaining further insight into skeletal muscle health.

In addition to exploring the screening potential of HGS asymmetry, there is also a strong need to elucidate its aetiological origins. While our understanding of muscle strength regulation has evolved over recent years, with neural and neuromuscular processes emerging as critical contributors [18–20], the regulatory mechanisms underpinning muscle strength symmetry are less understood. There is, however, emerging evidence to suggest that neuromuscular processes are likely contributors [16, 21]. Therefore, although a degree of HGS asymmetry is somewhat expected due to inherent hand preferences for completing habitual tasks (frequently reported as ~ 10%) [15, 22–24], having a difference > 10% may indicate a systemic deficit in neuromuscular function. Accordingly, we speculated that HGS asymmetry would correlate with increased circulating concentrations of neural and neuromuscular health markers. Specifically, for the present study we selected neural cell adhesion molecule (NCAM) as a marker of denervation [25], neurofilament light chain (NfL) as a marker of axonal integrity [26], and C-terminal agrin fragment (CAF) as a marker of neuromuscular junction stability [27]. Determining the association between these markers and HGS asymmetry may enhance our understanding of the mechanistic pathways underlying muscle strength symmetry and help guide future therapeutic interventions.

With the above in mind, the primary purpose of this study was to examine the relationship between HGS asymmetry and sarcopenia signatures in a large, well-characterised adult cohort. The secondary aim was to explore whether HGS asymmetry was associated with circulating concentrations of NCAM, NfL and CAF.

## Methods

### Study sample and sub-group analyses

Participants were recruited through the ‘GenoFit’ study, a large cross-sectional analysis of the Irish population that took place between September 2017 and October 2020 aiming to establish the relationship between genetics, fitness, lifestyle and health [3, 28]. In total, 10,546 individuals attended a single visit to one of two assessment clinics, during which a comprehensive panel of biometric and lifestyle data was collected. To be eligible for the present study subjects had to be ≥ 18 years of age, free from any musculoskeletal injury/impairment that may affect HGS (including hand, wrist or arm injuries, peripheral neuropathies such as carpal tunnel syndrome), free from any severe cognitive disorder and willing to provide written informed consent. Accordingly, the sample for the present study was refined to include 9403 participants aged between 18–92 years (males n = 4030 and females n = 5373). The primary analysis (n = 9403) examined differences in skeletal muscle index (SMI) and maximal HGS between those with symmetric and asymmetric HGS. The relationship between HGS asymmetry and signatures of sarcopenia (low maximal HGS, low SMI and sarcopenia) was examined in those aged ≥ 50 years (males n = 1168 and females n = 2316). The threshold of ≥ 50 years was chosen as we previously observed a marked deterioration in muscle health in this cohort from this age [3], and so deemed it to be most suited to assess sarcopenia related phenotypes. Plasma concentrations of NCAM, NfL and CAF were determined in a subgroup of 269 randomly selected individuals aged between 50–83 years (males n = 131 and females n = 138, full characteristics presented in Supplementary Table 1). Ethical approval was granted by the Human Research Ethics Committee, University College Dublin.

### Anthropometry and body composition analysis

Height and body mass were assessed using a SECA stadiometer and weighing scales (SECA, Hamburg, Germany) with participants dressed lightly and without footwear. Body mass index (BMI) was calculated as body mass divided by height squared (kg/m^2^). Dual energy x-ray absorptiometry (GE-LUNAR iDXA, Aymes Medical) was used to determine appendicular lean mass (the sum of lean mass of the limbs). Skeletal muscle index was defined as appendicular lean mass divided by height squared (kg/m^2^).

### Maximal HGS

Handgrip strength was determined using a digital hand-held dynamometer (Jamar, JLW Instruments, Chicago, IL, USA) according to a previously described protocol [3]. Following an explanation of the protocol and adjustment of the dynamometer for hand size, each participant completed two maximal efforts with each hand (~ three seconds per attempt) while in a standing position with their arm straight by their side. The highest recorded HGS value (regardless of hand dominance) was considered in the analysis.

### HGS asymmetry classification

Handgrip strength asymmetry was determined using a previously described approach [6]. Briefly, the highest recorded HGS value (irrespective of hand dominance) was divided by the highest recorded HGS of the opposite hand. The ‘10% rule’ was then applied, meaning that individuals with ratios > 1.10 were classified as having asymmetrical HGS, while those with a ratio of 1–1.10 were classified as having symmetrical HGS.

### Sarcopenia classification

Sarcopenia was diagnosed according to the European Working Group on Sarcopenia in Older People recommendations [10]. Cohort specific cut-off thresholds were established as 2 SDs below the mean of a young reference group (aged 20–39 years, males n = 1833 and females n = 1702). Accordingly, low HGS was defined as < 33.96 kg and < 21.66 kg for males and females, respectively, while low SMI was classified as < 7.27 kg/m^2^ and < 5.44 kg/m^2^ for males and females, respectively. Sarcopenia was diagnosed upon the presence of both low HGS and low SMI. Individual sarcopenia domains (low HGS only and low SMI only) were also considered in the analyses. Of those aged ≥ 50 years, 66 had sarcopenia (males n = 25, females n = 41), 244 had low HGS only (males n = 74, females n = 170) and 96 had low SMI only (males n = 45, females n = 51).

### Blood sampling and determination of plasma NCAM, NfL and CAF concentrations

Blood samples were collected by venepuncture of the median cubital vein and EDTA vacutainers (BD Vacutainer®). Samples were rested for 30 min and then centrifuged at 4000×*g* for 10 min at 4 °C. Plasma was subsequently pipetted into aliquots and immediately stored at -80 °C until time of analysis. Plasma concentrations of NfL and CAF were determined by ELISA (#OKCD01380, Aviva Systems Biology, San Diego, CA, USA and #ab216945, Abcam, Cambridge, UK, respectively) according to previously described protocols [18, 19]. Plasma NCAM levels were also measured using a commercially available ELISA (#ab119587, Abcam, Cambridge, UK) according to the manufacturer’s instructions. Briefly, 100 μL of standards and diluted sample (100-fold dilution) were added to the pre-coated microplate and incubated at 37 °C for 90 min. Then, 100 μL of NCAM detector antibody was added to each well and incubated at 37 °C for 60 min. Next, the microplate was washed three times and then 100 μL of Avidin–Biotin-Peroxidase Complex working solution was added into each well and incubated at 37 °C for a further 30 min. The microplate was washed five times and then Add 90 μL of TMB was added into each well and incubated at 37 °C for 20 min in the dark. Lastly, 100 μL of TMB stop solution was added into each well and the measurements were read at 450 nm (CLARIOStar BMG Labtech Microplate reader).

### Covariates

Educational attainment, smoking status, alcohol consumption, the prevalence of diseases/disorders and habitual physical activity were assessed using a self-reported questionnaire [3]. Educational attainment was determined by asking: "What is the highest level of education you have completed to date (no formal education, primary, lower secondary, higher secondary, third level, or postgraduate)?" Smoking status was classified as: (1) never smoked (never smoked/smoked < 100 cigarettes in lifetime), (2) previous smoker (smoked ≥ 100 cigarettes in lifetime but has now stopped smoking), and (3) current smoker (smoked ≥ 100 cigarettes in lifetime and currently smoking). Alcohol consumption was established by asking: “On average, how many standard drinks do you drink per week?" The presence of 56 diseases/disorders (cancers, heart diseases/disorders, skin disorders, digestive and bowel disorders, breathing disorders, bone and joint disorders, pain disorders, mental health conditions, brain/neurological disorders and diabetes; full list can be seen in Supplementary Material) was determined by asking: "Have you ever received a medical diagnosis from a doctor for any of the following conditions?" Finally, physical activity level was quantified by asking: "How many days per week do you do at least 30 min of moderate-intensity exercise that increases your breathing and heart rate (e.g. brisk walking, jogging, cycling, swimming)?”.

### Statistical analyses

Data are presented as means ± standard deviations (SD) unless stated otherwise. Data were assessed for normality using residual plots, skewness data and kurtosis data. The data were normally distributed. Individual samples Student's T-test and Chi-square tests were used to examine differences between those with HGS symmetry and HGS asymmetry for continuous and categorical variables. Multiple linear regression was used to assess the association between HGS asymmetry, maximal HGS and SMI. Analysis of covariance (ANCOVA) was used to assess differences in SMI and maximal HGS between those with symmetric and asymmetric HGS. Binary logistic regression was used to determine the odds for sarcopenia, low HGS only and low SMI only according to the presence of asymmetric HGS. Linear regressions, binary logistic regressions and ANCOVAs were adjusted for potential confounding factors including sex, age, BMI, disease prevalence (total number of diseases present), activity levels, smoking status, education, and alcohol consumption. Receiver operating characteristic (ROC) analyses were performed to determine the diagnostic utility of HGS asymmetry for sarcopenia, low HGS only and low SMI only. Corresponding optimal cut-off thresholds were determined by Youden’s index. All statistical analyses were performed using the SPSS software (Version 27, IBM SPSS Inc., Chicago, Il, USA) with the statistical significance threshold set at p < 0.05. Data visualisations were created using Prism (Version 9.3.1, GraphPad, Prism, San Diego, CA, USA).

## Results

### Study sample

The overall characteristics of the study sample according to HGS asymmetry are presented in Table 1. A total of 9403 subjects aged between 18–92 years participated in this study (males n = 4030 and females n = 5373). Handgrip strength asymmetry was present in 3771 (40%) individuals. Those with asymmetric HGS were older, less physically active, had lower maximal HGS and SMI, and more disorders/diseases than those with symmetric HGS (Table 1).

**Table 1:** Participant characteristics according to handgrip strength (HGS) asymmetry

Parameter	Total (n = 9403)	HGS symmetry (n = 5632)	HGS asymmetry (n = 3771)	p-value
Age (years)	44.9 ± 13.3	44.3 ± 13.0	45.7 ± 13.7	< 0.001
*Age groups, n (%)*				
18–34 years	2494 (26.5)	1544 (27.4)	950 (25.2)	< 0.001
35–49 years	3426 (36.4)	2103 (37.3)	1323 (35.1)	
50–64 years	2729 (29.0)	1595 (28.3)	1134 (30.1)	
≥ 65 years	754 (8.0)	390 (6.9)	364 (9.7)	
Height (cm)	170.9 ± 9.5	171.0 ± 9.5	170.7 ± 9.5	0.164
Body mass (kg)	73.9 ± 14.2	73.9 ± 14.1	73.8 ± 14.2	0.928
Body mass index (kg/m^2^)	25.2 ± 3.7	25.1 ± 3.7	25.2 ± 3.7	0.353
HGS (kg)	38.5 ± 11.7	39.3 ± 11.8	37.3 ± 11.4	< 0.001
Skeletal muscle index (kg/m^2^)	7.7 ± 1.4	7.7 ± 1.4	7.6 ± 1.4	< 0.001
*Education, n (%)*				
No formal/primary education	72 (0.8)	44 (0.8)	28 (0.8)	0.601
Lower secondary	313 (3.3)	195 (3.5)	118 (3.1)	
Higher secondary	1270 (13.5)	746 (13.2)	524 (13.9)	
Third-level degree	5099 (54.2)	3088 (54.8)	2011 (53.3)	
Postgraduate degree	2649 (28.2)	1559 (27.7)	1090 (28.9)	
*Smoking status, n (%)*				
Never (< 100 cigarettes)	5598 (59.5)	3321 (59.0)	2277 (60.4)	0.110
Previous smoker (> 100 cigarettes)	1979 (21.1)	1226 (21.8)	753 (20.0)	
Current smoker (> 100 cigarettes)	1826 (19.4)	1085 (19.2)	741 (19.6)	
Alcohol consumption (units/wk)	6.6 ± 6.1	6.6 ± 6.2	6.5 ± 6.0	0.290
*No. of diseases/disorders, n (%)*				
None	3647 (38.8)	2212 (39.3)	1435 (38.1)	0.011
One	2842 (30.2)	1740 (30.9)	1102 (29.2)	
Two or more	2914 (31.0)	1680 (29.8)	1234 (32.7)	
Physical activity^a^	4.1 ± 2.1	4.2 ± 2.0	4.0 ± 2.1	0.024

^a^ = days per week performing ≥ 30 min moderate intensity exercise

### Association between HGS asymmetry, maximal HGS and SMI

Multiple linear regression revealed that HGS symmetry was negatively associated with maximal HGS and SMI (both p < 0.001) after adjustment for several relevant confounders (Supplementary Table 2). Differences in SMI and maximal HGS between those with symmetric HGS and asymmetric HGS are displayed in Table 2. Overall, individuals with asymmetrical HGS had significantly lower SMI (7.67 kg/m^2^ vs 7.71 kg/m^2^, *p* = 0.004) and lower HGS (37.82 kg vs 38.91 kg, p < 0.001) compared to those with symmetrical HGS. When stratified by sex, males with asymmetrical HGS had lower SMI (8.89 kg/m^2^ vs 8.95 kg/m^2^, *p* = 0.004) and lower HGS (48.29 kg vs 49.97 kg, p < 0.001) compared to males with symmetrical HGS. In the female population, those with asymmetrical HGS had significantly lower HGS (29.93 kg vs 30.61 kg, p < 0.001) and marginally lower, albeit non-significant SMI (6.75 kg/m^2^ vs 6.78 kg/m^2^, *p* = 0.136), than females with symmetrical HGS.

**Table 2:** Differences in maximal handgrip strength (HGS) and skeletal muscle index (SMI) between those with symmetric and asymmetric HGS

Phenotype	Symmetric HGS (n = 5632)	Asymmetric HGS (n = 3771)	p-value
*SMI (kg/m*^*2*^*)*			
Total^a^	7.71 (0.01)	7.67 (0.01)	0.004
Males^b^	8.95 (0.01)	8.89 (0.02)	0.004
Females^b^	6.78 (0.01)	6.75 (0.01)	0.136
*HGS (kg)*			
Total^a^	38.91 (0.09)	37.82 (0.11)	< 0.001
Males^b^	49.97 (0.16)	48.29 (0.21)	< 0.001
Females^b^	30.61 (0.09)	29.93 (0.11)	< 0.001

Data presented as mean (standard error of mean); ^a^ = Adjusted for sex, age, body mass index, number of diseases present, activity level, smoking status, educational attainment, alcohol consumption; ^b^ = model ^a^ without sex; symmetric HGS males n = 2495, females n = 3137; asymmetric HGS males n = 1535, females n = 2236

### Risk of sarcopenia and individual sarcopenia signatures according to HGS asymmetry

Odds for sarcopenia, low HGS only and low SMI only, according to the presence of asymmetric HGS in individuals aged ≥ 50 years are presented in Table 3. Overall, those with asymmetrical HGS had significantly higher odds for sarcopenia (OR = 2.665, 95% CI 1.557–4.561, p < 0.001), low HGS (OR = 1.828, 95% CI 1.427–2.342, p < 0.001) and low SMI (OR = 1.792, 95% CI 1.257–2.554, *p* = 0.001), than those with symmetrical HGS. In the male population, HGS asymmetry was associated with significantly greater odds for sarcopenia, low HGS and low SMI, whereas in females greater odds were observed for sarcopenia and low HGS, but not low SMI (Table 3).

**Table 3:** Odds for sarcopenia and individual sarcopenia signatures according to the presence of asymmetric HGS in those aged ≥ 50 years

Domain	Minimally-adjusted ^a^ OR (95% CI)	p-value	Fully-adjusted ^b^ OR (95% CI)	p-value
*Sarcopenia (n* = *66)*				
Total	2.563 (1.518 – 4.328)	< 0.001	2.665 (1.557 – 4.561)	< 0.001
Males	3.728 (1.527 – 9.100)	0.004	3.334 (1.334 – 8.330)	0.010
Females	2.062 (1.076 – 3.951)	0.029	2.308 (1.177 – 4.525)	0.015
*Low handgrip strength only (n* = *244)*				
Total	1.817 (1.422 – 2.321)	< 0.001	1.828 (1.427 – 2.342)	< 0.001
Males	1.633 (1.063 – 2.508)	0.025	1.655 (1.069 – 2.562)	0.024
Females	1.911 (1.417 – 2.576)	< 0.001	2.014 (1.479 – 2.742)	< 0.001
*Low skeletal muscle index only (n* = *96)*				
Total	1.633 (1.175 – 2.271)	0.004	1.792 (1.257 – 2.554)	0.001
Males	1.869 (1.122 – 3.112)	0.016	1.925 (1.101 – 3.368)	0.022
Females	1.491 (0.967 – 2.298)	0.071	1.461 (0.731 – 2.918)	0.283

^a^Adjusted for sex (only in total groups), age and body mass index

^b^Adjusted for sex (only in total groups), age, body mass index, number of diseases present, activity level, smoking status, educational attainment, alcohol consumption; total sample n = 3483

### ROC analysis

**Fig. 1:**
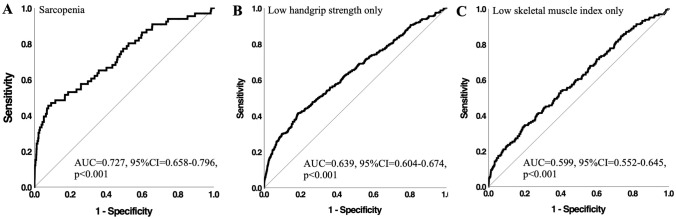
Receiver operating characteristic curves of handgrip strength (HGS) asymmetry for: **A** sarcopenia, **B** low HGS only and **C** low skeletal muscle index only

Figure 1 displays ROC curves of HGS asymmetry for sarcopenia and individual sarcopenia signatures. Level of HGS asymmetry demonstrated acceptable diagnostic accuracy for sarcopenia with an area under the curve (AUC) of 0.727 (95% CI 0.658–0.796, *p* < 0.001) and corresponding optimal HGS asymmetry ratio of 1.24 (45.5% sensitivity and 92.3% specificity). For low HGS only and low SMI only, the AUCs were 0.639 (95% CI 0.604–0.674, p < 0.001) and 0.599 (95% CI 0.552–0.645, p < 0.001), respectively. The optimal HGS asymmetry ratio was 1.17 for low HGS and low SMI, with corresponding sensitivity values of 41.6% and 34.6% and specificity values of 81.2% and 79.9%.

### Association between HGS asymmetry and plasma concentrations of NCAM, NfL and CAF

**Fig. 2:**
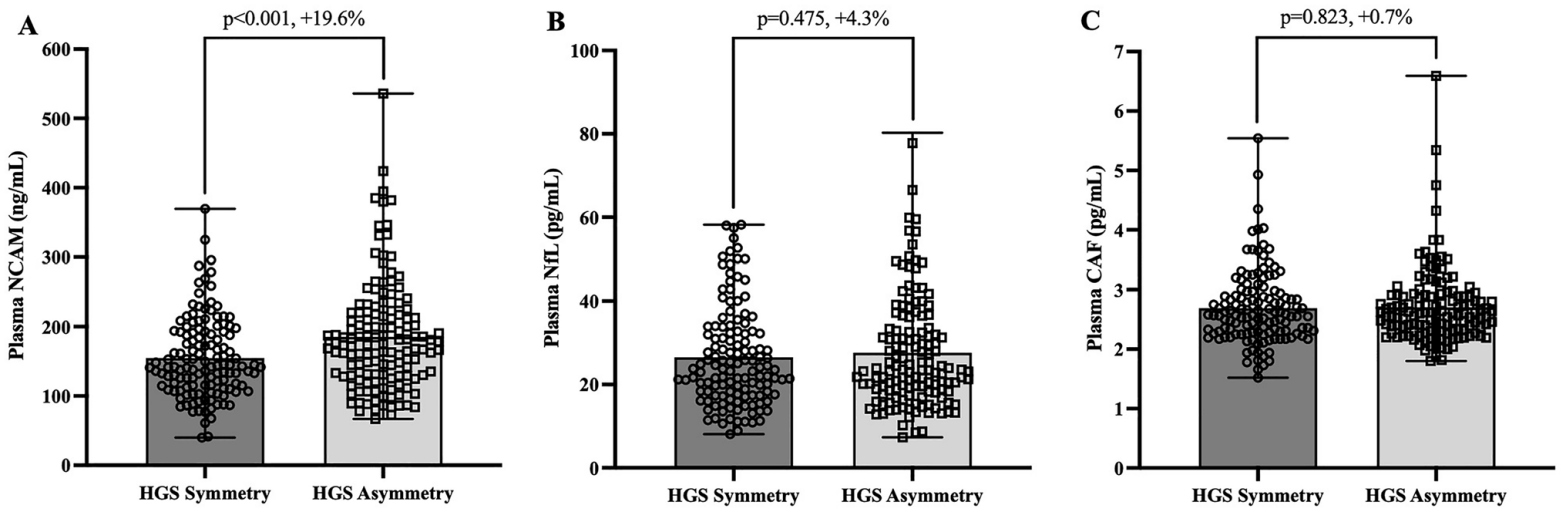
Plasma concentrations of neural and neuromuscular health markers according to HGS symmetry in 269 subjects aged between 50 and 83 years (A = NCAM: neural cell adhesion molecule, B = NfL: neurofilament light chain, C = CAF: C-terminal agrin fragment, HGS: handgrip strength, HGS symmetry n = 130, HGS asymmetry n = 139)

Figure 2 showcases the differences in circulating concentrations of NCAM, NfL and CAF according to HGS asymmetry. Plasma NCAM concentrations were 19.6% higher in individuals with asymmetrical HGS, compared to those with symmetrical HGS (185.40 ng/mL vs 155.00 ng/mL, p < 0.001). Notably, this association withstood adjustment for sex, age, BMI, number of diseases present and physical activity level (13.9% higher in those with asymmetrical HGS; 182.82 ng/mL vs 160.54 ng/mL, *p* = 0.016). Plasma NfL and CAF concentrations were 4.3% and 0.7% higher, respectively, in those with asymmetrical HGS compared to symmetrical HGS, however these differences did not reach statistical significance (27.65 pg/mL vs 26.52 pg/mL, *p* = 0.475 and 2.71 pg/mL vs 2.69 pg/mL, *p* = 0.823).

## Discussion

This is the first study to showcase the relevance of HGS asymmetry to signatures of sarcopenia. Collectively, our findings provide evidence that supports the potential utility of HGS symmetry as a screening tool that may complement existing strategies to combat skeletal muscle degradation. In this regard, expanding current HGS assessment protocols to incorporate a measure of asymmetry may represent an attractive means of enhancing the prognostic capacity of HGS for sarcopenia screening.

Our findings support existing studies relating HGS asymmetry to functional outcomes [6, 15, 17] and further extend its screening relevance to include sarcopenia and dichotomisations of sarcopenia (low SMI only and low HGS only). Indeed, individuals with HGS asymmetry had significantly lower SMI and lower HGS than those with symmetrical HGS. Moreover, we demonstrate those with asymmetric HGS to have 2.67 greater odds for sarcopenia, 1.83 greater odds for low HGS only and 1.79 greater odds for low SMI only, compared to subjects with symmetric HGS. The associations between HGS asymmetry and odds for individual sarcopenia signatures (low HGS only and low SMI only) are particularly encouraging as support for the potential of HGS asymmetry as a screening tool for muscle health. Nevertheless, the ROC analyses revealed HGS asymmetry to have acceptable diagnostic accuracy only for sarcopenia, and so additional studies are needed to confirm the diagnostic utility of HGS asymmetry for individual sarcopenia signatures. Furthermore, the corresponding optimal HGS asymmetry ratio for diagnosing sarcopenia was 1.24, which is notably higher than the > 10% threshold frequently used to classify asymmetric grip strength. Therefore, future studies should seek to confirm the optimal diagnostic threshold for screening sarcopenia. Notwithstanding these areas requiring further research, current data provide compelling support to include a measure of asymmetry within routine HGS assessments. Doing so may enhance the screening utility of HGS for identifying individuals at a clinically relevant, early stage of muscle health, thus facilitating timely therapeutic strategies. Importantly, the inclusion of HGS asymmetry interpretation would not impede the current convenience of HGS assessment as the relevant data are routinely collected as part of existing HGS protocols [11, 12].

Although right handed individuals (~ 90% of the population [29]) are typically stronger in their dominant hand, the degree of asymmetry is considerably lower in left handed individuals [23]. Interestingly, when performing a spontaneous grasping task, right handed individuals exhibit a marked preference in using their right hand, whereas left handed subjects use right and left hands equally [30]. Such phenomena may help explain the greater degree of strength asymmetry typically observed in right handed subjects. To date, studies examining physiological differences between right and left handed individuals have provided some insight into potentially relevant pathways underpinning strength asymmetry. Although the precise mechanisms are yet to be fully elucidated, neural processes may be centrally involved [21, 31–33]. For example, right handed people exhibit asymmetrical surface area and activation of cortical areas, while in contrast, left handed people demonstrate more symmetric surface areas and activation patterns [31–33]. With this in mind, and considering HGS asymmetry is lower in left handed subjects, it is plausible that neural control is a relevant contributor to HGS asymmetry. Notably, the findings from the biomarker component of the present study support this.

Indeed, we found plasma concentrations of NCAM to be 19.6% higher in individuals with asymmetrical HGS compared to those with symmetrical HGS. This indicates a greater level of denervation in those with HGS asymmetry and aligns with existing data that suggest hand function and coordination to be intricately linked with neurological health [34–36]. Interestingly, ageing is generally accompanied by a natural shift towards ambidexterity [37], which hypothetically should promote HGS symmetry. In light of this, we propose that the presence of HGS asymmetry in older adulthood, notwithstanding the tendency to ambidexterity, may indicate a particular deterioration of neural health. In this regard, it is plausible that deficits in neural function disrupt the intricacy of proper hand function, and over time manifest into asymmetric HGS. Considering the presence of increased NCAM levels it would seem logical that concentrations of NfL and CAF, as markers of axonal damage [26] and NMJ integrity [27], would also be elevated. Perhaps surprisingly, in the present study plasma concentrations of NfL and CAF were not significantly different between groups. Nevertheless, despite not reaching significance, plasma NfL concentrations were 4.3% higher in those with HGS asymmetry, suggesting axonal damage may be a contributory factor. Notably, our group recently demonstrated that circulating NfL concentrations are associated with maximal HGS and sarcopenia [18], illuminating the relevance of axonal integrity to skeletal muscle health. Therefore, although the between-group differences in NfL concentrations did not achieve significance in this study, the potential interplay between neural health, HGS asymmetry and circulating NfL is promising and warrants further investigation.

The strengths of the present study include the large, well-characterised study sample, coupled with statistical adjustment for relevant covariates. Additionally, the incorporation of biomarker analyses provided insight into potentially relevant pathways underpinning HGS asymmetry and may help guide future studies. Several limitations to our work should also be acknowledged. Firstly, this study was cross-sectional, and so we cannot infer causality between variables. Secondly, while amendments to the GenoFit protocol were not possible in this instance, recording hand dominance would have been useful. Understanding the potential interplay between handedness, HGS asymmetry and sarcopenia signatures is important for the generalisability of our findings. Thirdly, using magnetic resonance imaging to determine muscle mass may have provided a better representation of sarcopenia, due to the inherent inaccuracies of DXA in underestimating the age-related decline of muscle mass [38]. Fourthly, assessing plasma NfL concentrations using a more sensitive analytical method such as single molecular array [39] may have been beneficial. Finally, although our study incorporated an age-diverse and nationally representative sample, the present study included only those residing in Ireland, and so the generalisability of our findings to other populations needs to be determined.

In conclusion, our study demonstrates the relevance of HGS asymmetry as a screening tool for sarcopenia. Specifically, we show that those with asymmetric HGS have significantly greater odds for sarcopenia, low HGS and low SMI, compared to those with symmetric HGS. Broadening current HGS assessment protocols to include a measure of asymmetry may enhance the prognostic value of HGS for identifying sarcopenia risk, and allow for a more rapid deployment of therapeutic strategies. Importantly, such expansion would not hinder the present convenience of routine HGS assessment as the required data are already collected as part of existing HGS protocols. The biomarker component of our work supports the relevance of neural health as a principal pathway underlying HGS asymmetry. Denervation appears to be a particularly likely contributor, while the potential relevance of axonal damage should be investigated further.

### Supplementary Information

Below is the link to the electronic supplementary material.

Supplementary file1 (DOCX 20 KB)

## Data Availability

Data may be made available upon reasonable request to the corresponding author.
