# Supplementary material for: Handgrip strength asymmetry as a new biomarker for sarcopenia and individual sarcopenia signatures
Source: Aging Clin Exp Res. 2023 Sep 2;35(11):2563–71. doi: 10.1007/s40520-023-02539-z (PMC10627945; doi:10.1007/s40520-023-02539-z)
Supplement: Supplementary file 1 — Supplementary file1 (DOCX 20 KB) [file 40520_2023_2539_MOESM1_ESM.docx]

| **Supplementary Table 1** Participant characteristics aged ≥50 years according to biomarker data availability | | | |
| --- | --- | --- | --- |
| Parameter | No biomarker data  (n=3214) | Biomarker data  (n=269) | p-value |
| Age (years) | 58.7 (6.5) | 65.5±8.4 | <0.001 |
| Height (cm) | 167.9±8.9 | 168.6±9.5 | 0.197 |
| Body mass (kg) | 72.7±13.9 | 73.9±13.6 | 0.191 |
| Body mass index (kg/m^2^) | 25.7±3.6 | 25.9±3.4 | 0.500 |
| HGS (kg) | 34.0±10.2 | 33.9±11.7 | 0.886 |
| Skeletal muscle index (kg/m^2^) | 7.3±1.2 | 7.4±1.3 | 0.088 |
| *Education, n (%)* |  |  |  |
| No formal/primary education | 50 (1.6) | 8 (3.0) | 0.091 |
| Lower secondary | 210 (6.5) | 23 (8.6) |  |
| Higher secondary | 626 (19.5) | 63 (23.4) |  |
| Third-level degree | 1644 (51.2) | 120 (44.6) |  |
| Postgraduate degree | 684 (21.3) | 55 (20.4) |  |
| *Smoking status, n (%)* |  |  |  |
| Never (<100 cigarettes) | 1740 (54.1) | 155 (57.6) | 0.097 |
| Previous smoker (>100 cigarettes) | 819 (25.5) | 47 (17.4) |  |
| Current smoker (>100 cigarettes) | 655 (20.4) | 67 (24.9) |  |
| Alcohol consumption (units/wk) | 7.2±6.4 | 7.1±6.9 | 0.841 |
| *Number of diseases/disorders, n (%)* |  |  |  |
| None | 947 (29.5) | 66 (24.5) | 0.088 |
| One | 960 (29.9) | 76 (28.3) |  |
| Two or more | 1308 (40.7) | 127 (47.2) |  |
| Physical activity ^a^ | 4.0±2.1 | 3.9±2.4 | 0.277 |
| ^a^ = days per week performing ≥ 30 minutes moderate intensity exercise | | | |

| **Supplementary Table 2** Association between handgrip strength (HGS) asymmetry, maximal HGS and skeletal muscle index (SMI) | | | | |
| --- | --- | --- | --- | --- |
|  | HGS symmetry ratio | | | |
| Phenotype | β | 95% CI | p-value | R^2^ |
| Maximal HGS ^a^ | -0.002 | -0.003 - -0.002 | <0.001 | 0.028 |
| SMI ^a^ | -0.005 | -0.009 - -0.002 | <0.001 | 0.010 |
| ^a^ = Adjusted for sex, age, BMI, number of diseases present, activity levels, smoking status, education and alcohol consumption; n = 9403 | | | | |

**List of diseases/disorders screened**

*A count for diseases/disorders present was computed for every participant.*

- Cancer: blood leukaemia, breast, colon, kidney, lung, ovarian, pancreas, prostate, skin
- Heart disease/disorder: abnormal heart beat arrhythmia, angina, heart attack, high blood pressure, high cholesterol, stroke
- Skin disorder: eczema, melasma, psoriasis, rosacea, scleroderma
- Digestive/bowel disorder: coeliac disease, Crohn’s disease, fatty liver disease, inflammatory bowel disease, irritable bowel syndrome, ulcerative colitis
- Breathing disorder: asthma, chronic obstructive pulmonary disease, emphysema, cystic fibrosis, idiopathic pulmonary fibrosis, lung fibrosis, sarcoidosis
- Bone/joint disorder: ankylosing spondylitis spondylarthritis, enteropathic arthritis, osteoarthritis, psoriatic arthritis, reactive arthritis, rheumatoid arthritis, osteoporosis
- Pain disorder: chronic back pain, migraine
- Mental health condition: anxiety, bipolar disorder, depression, schizophrenia
- Neurological: Alzheimer’s, age related macular degeneration, autism, epilepsy, motor neurone disease, multiple sclerosis, Parkinson’s
- Diabetes: gestational diabetes, type 1 diabetes, type 2 diabetes
